# The dying-moment dream hypothesis: heaven and hell as the brain’s final dream

**DOI:** 10.3389/fpsyg.2026.1766053

**Published:** 2026-03-25

**Authors:** Recai Kayış

**Affiliations:** Department of Psychology, Istanbul Aydın University, Istanbul, Türkiye

**Keywords:** consciousness, DMT, end-of-life visions, gamma oscillations, near-death experience, temporal distortion, terminal brain activity

## Abstract

Reports of near-death experiences (NDEs), end-of-life visions (ELVs), and culturally embedded afterlife narratives frequently describe profoundly positive or distressing states. Traditional interpretations treat these phenomena as evidence of external metaphysical realms. The Dying-Moment Dream Hypothesis proposes an alternative, neurobiologically grounded explanation: that culturally conditioned afterlife experiences may constitute a final, endogenous simulation (“dream”) generated by the dying brain. This simulation is hypothesized to emerge from a confluence of transiently heightened terminal neural activity, affective-memory integration, temporal processing collapse, and the brain’s neurochemical end-of-life cascade. Because subjective time may dilate under hypoxic stress, seconds of neural activity may be computationally experienced as a perceived loss of temporal boundaries. The absence of a subsequent awakening is proposed to render this final simulation the individual’s last phenomenologically accessible conscious state. This paper synthesizes evidence from prospective NDE studies, terminal EEG recordings, dream neuroscience, cultural cognition, psychedelic research, and hospice ethnography to present a unified theoretical framework. Limitations, competing models, and explicitly falsifiable predictions are discussed.

## 1 Introduction

Beliefs about the afterlife—particularly dichotomous concepts of reward and punishment—have been central to human cultures for millennia (Eliade, 1957). While theological frameworks posit these states as external realities, contemporary neuroscience offers increasingly detailed insight into the phenomenology of consciousness during the dying process. Prospective studies of cardiac arrest survivors suggest that conscious-like activity may persist even during apparent EEG flatline conditions (Parnia et al., 2023).

This article advances the Dying-Moment Dream Hypothesis, which asserts that the afterlife-like experiences reported near death represent a self-generated, final simulation produced by the dying brain. This experience is shaped by the individual’s emotional history, autobiographical memory, moral self-concept, and cultural schemas. Because temporal processing mechanisms degrade under hypoxia, the subjective experience may extend into a perceived loss of temporal boundaries. The hypothesis provides a naturalistic explanation for cross-cultural variability in afterlife imagery, the predominance of positive NDEs, and the phenomenological similarities between NDEs and intense dream or psychedelic states.

## 2 Theoretical background

### 2.1 Near-death experiences and end-of-life phenomenology

NDEs typically include features such as detachment from the body, panoramic memory, encounters with luminous beings, and entry into peaceful or terrifying environments (Greyson, 1983). ELVs in hospice patients frequently mirror personal emotional themes and unresolved relational dynamics (Menzies et al., 2022). These phenomena challenge simplistic biomedical models of brain shutdown, suggesting that some form of organized conscious activity may persist temporarily during physiological collapse.

### 2.2 Terminal brain function and neurodynamics

Recent investigations challenge the traditional assumption that cortical activity ceases rapidly and uniformly following cardiac arrest. Experimental and clinical observations instead suggest that early peri-arrest phases may involve transient, temporally organized high-frequency dynamics before complete electrical suppression.

In a controlled rodent model of asphyxial cardiac arrest, Borjigin et al. (2013) reported a marked transient increase in gamma-band oscillatory activity (≈25–55 Hz) within the first 30 s following oxygen deprivation. This surge was accompanied by elevated global coherence and increased phase–amplitude coupling between gamma oscillations and slower rhythms.

Some human clinical EEG recordings are consistent with these preclinical findings (Vicente et al., 2022; Xu et al., 2023). Analyzing electroencephalogram signals in comatose dying patients following the withdrawal of ventilatory support, Xu et al. (2023) identified a rapid and marked surge of absolute gamma power. Far from being a uniform burst of pathological noise, this high-frequency activity was predominantly observed in posterior regions including the temporo-parieto-occipital (TPO) junctions. In contemporary neuroscience, the TPO junctions constitute the posterior cortical “hot zone,” an anatomical hub widely implicated in conscious perception, visuospatial processing, dreaming, and out-of-body experiences (OBEs).

Beyond localized power surges, the dying human brain exhibits sophisticated hierarchical coordination. Xu et al. (2023) demonstrated elevated cross-regional phase-amplitude coupling (crPAC), where the phase of slower frequency bands (such as theta and alpha) modulated the amplitude of high-frequency gamma oscillations across different cortical areas, including interhemispheric interactions between the posterior hot zones and the contralateral prefrontal cortex. In healthy neurodynamics, such cross-frequency coupling is a widely recognized mechanism for cognitive binding, memory retrieval, and the integration of distributed neural networks into a unified conscious percept.

Furthermore, the directionality of this terminal information flow provides a critical mechanistic foundation for the Dying-Moment Dream Hypothesis. Utilizing advanced information-theoretic metrics such as Normalized Symbolic Transfer Entropy (NSTE), researchers have mapped the causal relationships and directed connectivity between electrical signals in the dying brain. Under normal waking conditions, perception is driven largely by feedforward (bottom-up) connectivity bringing sensory data from the external world. However, during terminal hypoxia, as external sensory input ceases, NSTE analyses reveal a marked, asymmetric increase in feedback (top-down) directed connectivity. When the brain is completely decoupled from external sensory constraints, it initiates a profound top-down projection of its own internal architecture—relying exclusively on memory and affective priors to generate a highly structured, closed-loop internal simulation.

**Figure 1:**
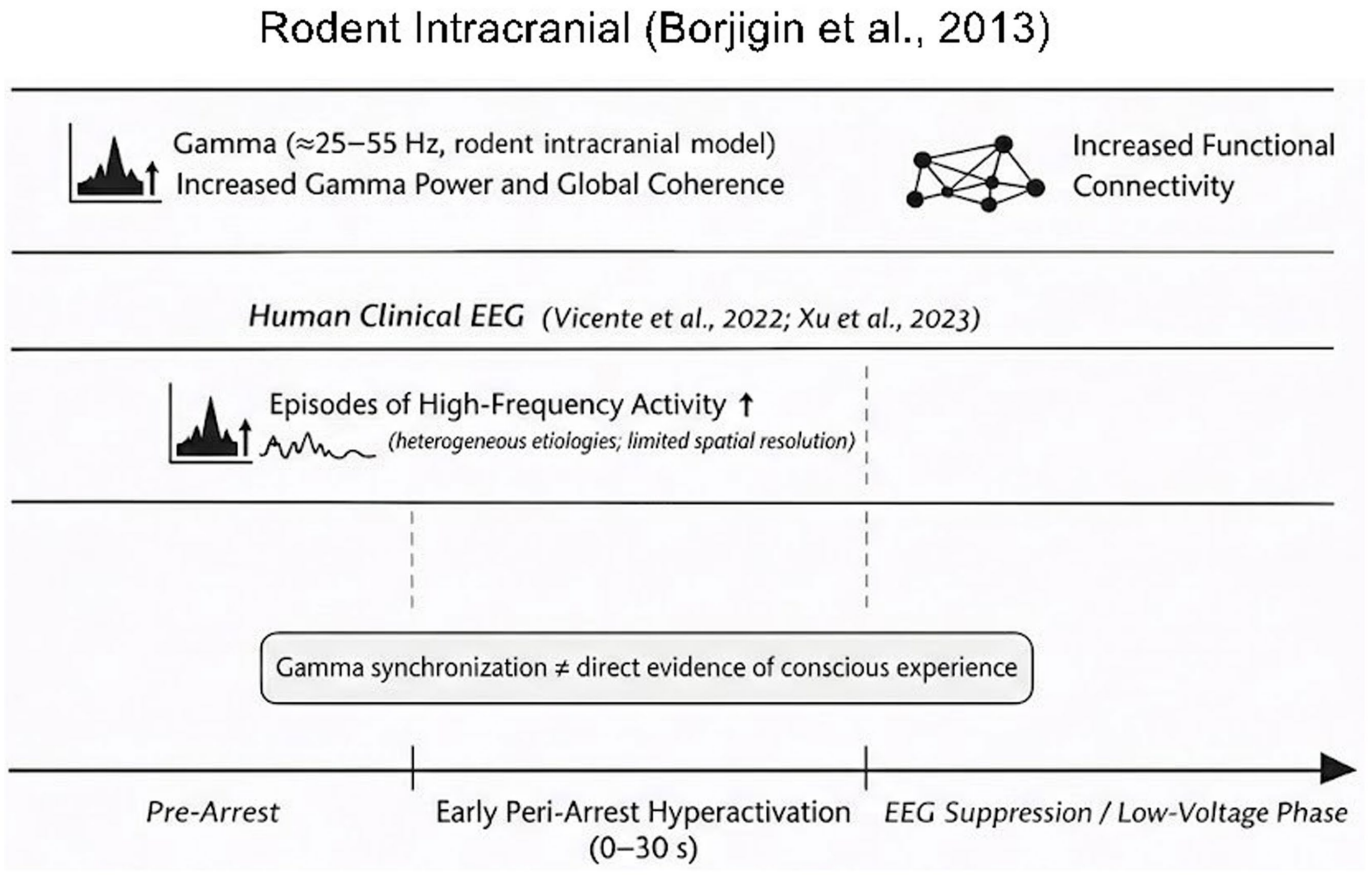
Model of surge in coherent neurophysiological activity in rodent intracrania I recordings (Borjigin et al., 2013) and human clinical EEG (Vicente et al., 2022; Xu et al., 2023) immediately following cardiac arrest, coinciding with reported NDE temporal window.

Despite these compelling metrics, epistemic caution remains necessary. High-frequency activity and network synchrony can also arise from transient reductions in inhibitory control (disinhibition) or excitotoxic cascades during metabolic collapse. Therefore, the present hypothesis does not assert that terminal gamma synchronization is incontrovertible proof of a sustained conscious state. Rather, it posits that the specific topographies (TPO hot zones), binding mechanisms (crPAC), and directed information flows (top-down NSTE) observed during the early peri-arrest window provide a neurobiologically plausible and highly permissive substrate for the generation of a complex, affectively driven final experience (see Figure 1 for a schematic summary of peri-arrest neurodynamics).

### 2.3 Dream neuroscience: limbic selection and the dream-lag effect

Dream research indicates that spontaneous mentation is not a replay of random imagery, but rather a highly structured integration of autobiographical memory, emotional salience, and unresolved concerns. Neuroimaging and electrophysiological studies of REM sleep consistently demonstrate heightened activity within limbic and paralimbic structures—particularly the amygdala and hippocampal complex—alongside marked reductions in dorsolateral prefrontal regulatory control (Hobson, 2009; Walker and van der Helm, 2009). To understand how the dying brain selects specific narrative themes (e.g., peace, judgment, or the panoramic life review) from a lifetime of memories, it is necessary to examine the mechanisms of sleep-dependent memory consolidation. A crucial phenomenon in this context is the “dream-lag effect,” characterized by a 7-day U-shaped temporal curve wherein waking experiences are incorporated into dreams immediately (day-residue) and then preferentially reappear 5 to 7 days later (Nielsen and Powell, 1989; Blagrove et al., 2011). Blagrove et al. (2011) demonstrated that this delayed incorporation is strictly specific to REM sleep and reflects the gradual transfer and consolidation of memory representations from the hippocampus to the neocortex.

Crucially, this consolidation process is not egalitarian. Dream-lag research shows that the brain does not preferentially incorporate major daily activities or routine events into dreams; rather, it selectively processes “Personally Significant Events” (PSEs) driven by high emotional intensity. The amygdala acts as a salience filter, tagging memories associated with survival, severe threat, trauma, or deep attachment.

**Figure 2:**
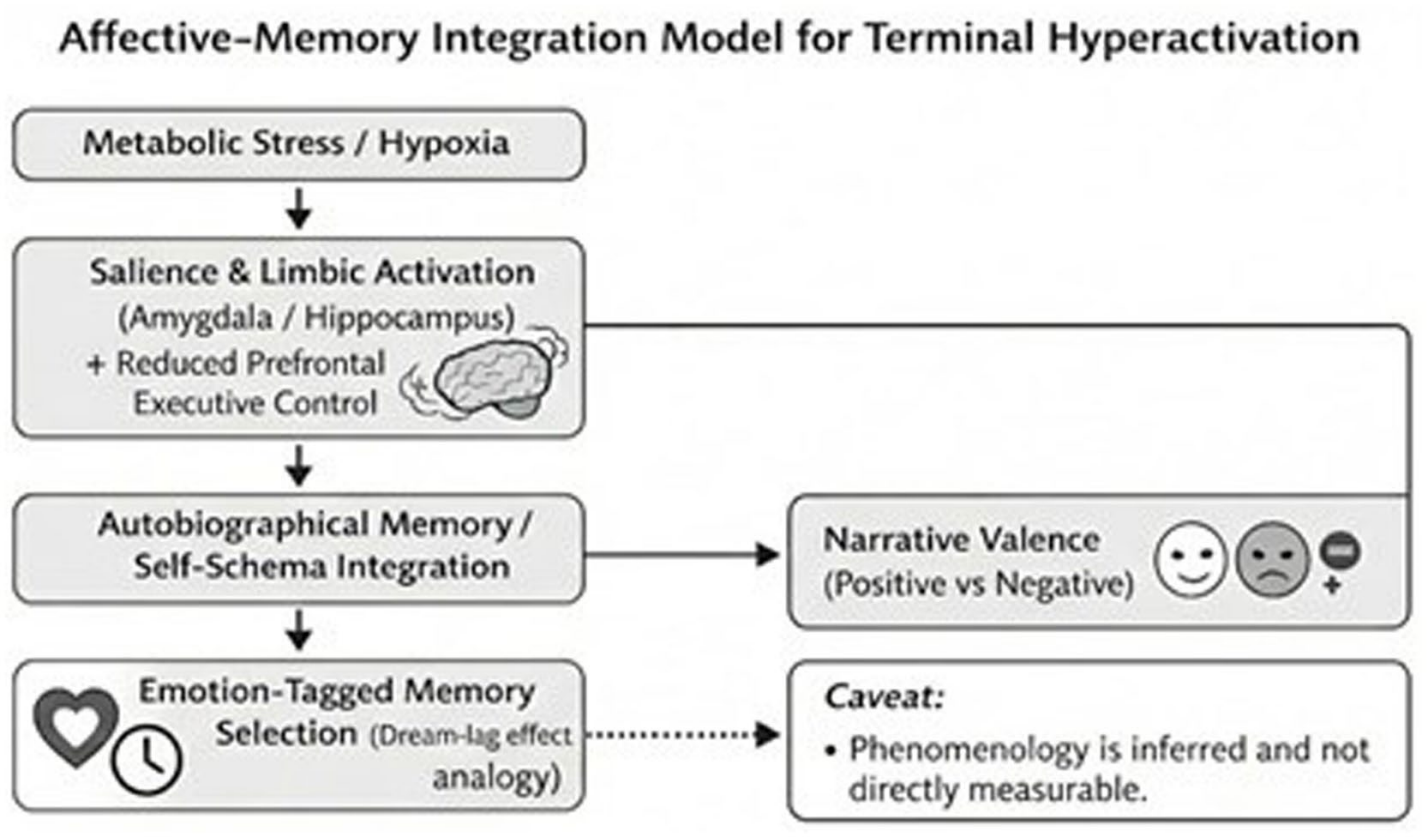
Hypothesized pathway linking terminal metabolic stress to limbic salience, reduced prefrontal control, and emotion-tagged autobiographical memory integration, yielding narratives with positive or negative valence.

Within the proposed framework, the terminal brain enters a severe hyper-associative mode under acute hypoxic stress and reduced top-down executive constraint. Here, the term “hyper-REM” is used strictly as a descriptive and metaphorical shorthand to denote a REM-like network configuration under acute metabolic crisis. It does not imply the existence of a distinct physiological sleep stage, nor does it suggest that the dying brain enters canonical REM sleep. Rather, it refers to a functional pattern characterized by limbic predominance, reduced prefrontal executive regulation, and intensified internally generated imagery. The acute metabolic crisis acts as a catalyst, rapidly and uncontrollably triggering the hippocampal-neocortical memory consolidation network. Because the prefrontal cortex—which normally exerts inhibitory control over the limbic system—is compromised, the amygdala’s emotional selection mechanism dominates. The brain hyper-associatively retrieves those memories carrying the highest emotional intensity (personally significant events) rather than objective chronological history. If an individual harbors unresolved moral guilt, deep-seated fear, or trauma, this disinhibited limbic hyperfunction selectively releases these high-intensity negative traces, constructing the phenomenological architecture of a distressing NDE. Conversely, a predominance of secure attachments and positive affective schemas generates comforting narratives. Thus, the emotional and moral “residue” of a person’s life forms the precise neurobiological script for their final simulation. This proposed affective-selection mechanism is summarized schematically in Figure 2.

### 2.4 Temporal distortion and predictive processing: the illusion of boundless duration

Accurate temporal perception is not a passive reception of objective time but an active, computational construct depending on distributed fronto-parietal and cortico-striatal networks. Neurocognitive models of interval timing implicate coordinated interactions among the dorsolateral prefrontal cortex, inferior parietal cortex, and basal ganglia in maintaining temporal order and subjective duration tracking. Disruption within these systems is consistently associated with time dilation, compression, or loss of temporal continuity.

To understand how a dying brain could subjectively experience a fleeting peri-arrest window as an extensive duration, this hypothesis incorporates the Predictive Processing (PP) framework. Under normal waking conditions, the brain functions as a hierarchical prediction engine, constantly generating top-down priors (predictions) that are continuously constrained and updated by bottom-up sensory prediction errors (external data). The subjective flow of time is anchored by the regular rhythmic updating of these external sensory inputs.

However, during cardiac arrest and terminal hypoxia, exteroceptive and interoceptive sensory inputs are abruptly severed. Applying the principles of the REBUS (RElaxed Beliefs Under pSychedelics) model to the terminal state, this profound deafferentation relaxes the “precision weighting” of high-level priors. Bereft of bottom-up sensory constraints, the brain’s internal generative models run unchecked. As inhibitory control fails, the amygdala-driven emotional memories flood the conscious workspace.

Because the brain estimates subjective duration based on the “event-rate” of neural information processing (the density of perceptual and cognitive changes), the chaotic, hyper-associative flood of internal imagery during the terminal gamma surge creates an extreme temporal dilation. A few seconds of objective, hyper-accelerated neural activity—packed with a lifetime of consolidated emotional memories—are computationally stretched. Consequently, the individual experiences a profound subjective illusion of boundless duration, often metaphorically described as “eternity” by experiencers.

### 2.5 Endogenous psychedelic mechanisms: the threshold debate

The structural similarities between classic serotonergic psychedelic experiences and NDEs have led to the hypothesis that the endogenous release of N,N-dimethyltryptamine (DMT) during the dying process triggers the final dream. However, this hypothesis has faced substantial neuropharmacological criticism. Nichols (2018) argues that while DMT is endogenously produced, it exists only in trace amounts and is rapidly degraded by ubiquitous monoamine oxidase (MAO) enzymes. According to this critique, the human pineal gland is too small and its biosynthetic capacity too limited to produce the massive quantities of DMT required to overcome MAO degradation and breach the “psychedelic threshold” necessary to induce a global altered state of consciousness.

While Nichols’ critique holds true for a healthy, metabolically stable brain, recent molecular findings offer a more nuanced, localized mechanism that supports the Dying-Moment Dream Hypothesis. Dean et al. (2019) demonstrated that the enzymes required for DMT biosynthesis—aromatic-L-amino acid decarboxylase (AADC) and indolethylamine-N-methyltransferase (INMT)—are robustly co-localized within the same neurons in the mammalian cerebral cortex and hippocampus. This co-localization indicates that DMT does not need to be synthesized exclusively in the pineal gland and transported globally; rather, it can be synthesized locally within the very cortical networks responsible for visuospatial perception and memory consolidation. Furthermore, Dean et al. (2019) reported a significant extracellular surge of DMT specifically in the visual cortex following experimental cardiac arrest, independent of an intact pineal gland.

Because there is currently no evidence that endogenous DMT reaches the required psychedelic threshold in the human brain, the present model conceptualizes DMT strictly as a permissive neuromodulator or co-factor rather than the primary causal driver of the experience. During cardiac arrest, severe hypoxia may induce a state of cortical disinhibition, reducing top-down regulatory control and increasing receptor sensitivity. In this hyper-excitable microenvironment, even minor, locally synthesized surges of endogenous DMT within the temporo-parieto-occipital (TPO) hot zones may function as a permissive amplifying factor, potentially facilitating the highly associative imagery of the final state.

## 3 Methodological framework: a scoping review approach

To enhance transparency, this theoretical synthesis was conceptually informed by selected reporting principles from the PRISMA-ScR framework (Tricco et al., 2018). However, it was not conducted as a fully systematic scoping review with exhaustive database coverage, formal screening stages, or preregistered protocols. Rather, PRISMA-ScR elements were used to structure the organization and reporting of interdisciplinary evidence.

### 3.1 Search strategy and information sources

A comprehensive literature search was conducted across major scientific databases, including PubMed/MEDLINE, Scopus, Web of Science, and PsycINFO. The search strategy utilized Boolean operators to combine terms related to the core phenomena and underlying mechanisms. Example search strings included: ((“near-death experience” OR “NDE” OR “end-of-life vision”) AND (“EEG” OR “gamma oscillations” OR “cardiac arrest” OR “resuscitation”)), ((“predictive processing” OR “time perception”) AND (“hypoxia” OR “psychedelic” OR “DMT”)), and (“dream-lag effect” OR “REM sleep” AND “memory consolidation”). While comprehensive, the literature search was not systematically exhausted across all dates or databases, focusing primarily on conceptually pivotal studies to construct the theoretical framework.

### 3.2 Eligibility criteria

Sources were selected based on the following inclusion criteria: (1) peer-reviewed empirical studies, clinical case reports, and theoretical models published in English; (2) studies directly measuring terminal neurodynamics (e.g., continuous peri-arrest EEG); (3) prospective clinical studies on cardiac arrest survivors (e.g., the AWARE trials); (4) neurobiological studies of dreaming and psychedelics mapping onto NDE phenomenology; and (5) structured cross-cultural comparative analyses. Studies consisting solely of unverified anecdotal accounts, non-peer-reviewed literature, or those lacking a rigorous empirical or philosophical framework were excluded from the primary synthesis.

### 3.3 Data charting and synthesis of results

Extracted data were not meta-analyzed due to the profound heterogeneity of the study designs (e.g., animal intracranial EEG versus human qualitative surveys). Instead, a conceptual synthesis method was employed. Data were charted into distinct thematic domains: terminal electrophysiology (gamma surges, PAC, directed connectivity), affective memory mechanisms (amygdala salience, dream-lag effect), predictive processing (temporal distortion), endogenous neuromodulation (DMT), and cultural phenomenology. These synthesized domains were then integrated to construct the multicomponent neurocognitive model presented in Section 4.

## 4 The dying-moment dream hypothesis: a multicomponent model

Rather than positing a single localized neural event, the Dying-Moment Dream Hypothesis is structured as a multicomponent neurocognitive model. It proposes that afterlife-like phenomenology is the experiential correlate of a specific, sequential breakdown of brain networks. The model synthesizes the empirical mechanisms detailed above into four interlocking propositions:

### 4.1 Terminal disinhibition and the endogenous simulation

As cerebral hypoxia initiates a metabolic crisis, the brain does not uniformly transition to electrical silence. Instead, the preferential failure of inhibitory interneurons induces a transient state of massive cortical disinhibition. This may trigger a localized surge of high-frequency gamma activity within the posterior temporo-parieto-occipital (TPO) hot zones, structurally bound by cross-regional phase-amplitude coupling (crPAC). Stripped of bottom-up sensory input, the brain’s directed connectivity shifts entirely to a top-down architecture (NSTE). Functionally, this neurodynamic configuration produces a closed-loop, hyper-associative simulation generated entirely from within the brain’s own architecture.

### 4.2 Affective-mnemonic scripting: the role of “residue”

The narrative content of this terminal simulation is not random. Driven by the amygdala’s salience network—analogous to the selective emotional memory consolidation seen in the sleep-dependent dream-lag effect—the disinhibited brain hyper-associatively retrieves “Personally Significant Events.” The individual’s deepest affective and moral history acts as the script. High-intensity emotional memories (unresolved guilt, trauma, secure attachments, and profound love) combined with culturally conditioned semantic priors (e.g., specific religious iconography) dictate the valence of the experience, manifesting phenomenologically as either distressing or comforting imagery.

### 4.3 Temporal decoupling and subjective time dilation

As fronto-parietal and cortico-striatal timing networks degrade, the brain loses its capacity for objective duration tracking. Within the predictive processing framework, the sudden loss of sensory prediction errors relaxes the precision weighting of the brain’s internal clock. Time perception shifts entirely to an “event-rate” metric. Because the terminal gamma surge packs a massive density of affective and mnemonic data into a narrow biological window, these final seconds may be computationally stretched. Subjectively, the experience dilates, creating a profound illusion of boundless duration.

### 4.4 The finality of the phenomenological state

In normal sleep architecture, a dream concludes upon the restoration of waking consciousness, allowing the brain to retroactively recognize the experience as an internally generated illusion. In the context of irreversible biological death, however, this reintegration never occurs. Without a subsequent waking state to falsify the simulation, the dying-moment dream remains the last conscious experience of the organism. Epistemologically and phenomenologically, this terminal simulation may constitute the final experiential state accessible to the organism.

## 5 Empirical convergence

### 5.1 Terminal EEG and gamma surges

Reports of peri-arrest and late bursts of structured electrophysiological activity complicate the long-standing assumption of rapid and uniform cortical shutdown following circulatory collapse. In animal models, Borjigin et al. (2013) reported increased gamma-band activity (≈25–55 Hz) and elevated coherence within the early peri-arrest window, alongside cross-frequency coupling patterns consistent with temporally structured organization. Human clinical EEG reports obtained during withdrawal of life-sustaining treatment describe episodes of increased high-frequency activity and inter-regional synchronization (Vicente et al., 2022; Xu et al., 2023). However, structured high-frequency activity does not, by itself, demonstrate preserved consciousness. Within the present framework, such transient organization is treated as a potentially permissive but not sufficient condition for the possibility of organized subjective experience.

### 5.2 Prospective NDE studies and phenomenological categorization

The AWARE-II study (Parnia et al., 2023) represents the most comprehensive prospective investigation of consciousness and its underlying electrocortical biomarkers during cardiopulmonary resuscitation (CPR). AWARE-II reported that despite marked cerebral ischemia (mean rSO2 = 43%), intermittent organized low-frequency activity (delta/theta/alpha bands) could be detected in some cases during prolonged resuscitation efforts, as late as 35 to 60 min into ongoing CPR.

Crucially, the study went beyond detecting “consciousness” by applying rigorous qualitative categorization. Among the 28 interviewed survivors from the in-hospital cardiac arrest cohort, 39.3% reported memories suggestive of consciousness. Related findings have also been reported in non-life-threatening events and coma of different etiologies (Charland-Verville et al., 2014). These experiences were distinct and phenomenologically categorized as shown in Table 1.

**Table 1:** Phenomenological categorization of recalled experiences during cardiac arrest (adapted from Parnia et al., 2023).

Experience category	Characteristics and NDE relevance	Prevalence (n = 28)
Transcendent recalled experience of death (RED)	Authentic near-death experiences featuring high narrative coherence, life review, and transcendent elements.	21.4% (6 patients)
Dream-like experiences	Visionary or hallucinatory experiences lacking the specific transcendent criteria of RED.	10.7% (3 patients)
CPR-induced consciousness (CPRIC)	Emergence from coma during CPR, perceiving the medical environment.	7.1% (2 patients)
Post-resuscitation period recall	Memories forming during the recovery phase rather than deep arrest.	7.1% (2 patients)
Delusions (misattribution)	Identified in the broader community cohort; misinterpreting medical events (e.g., CPR pain) as torture or hellish scenarios.	N/A (cross-sectional)

Furthermore, the explicit identification of a “Delusions” category—where external somatic stimuli (such as chest compressions or defibrillation) are incorporated and misattributed as internal, often terrifying narratives—is highly consistent with established mechanisms of dreaming. Just as a sleeping brain incorporates external stimuli into a dream narrative, the dying brain may integrate the traumatic sensory inputs of CPR with the patient’s affective state. Finally, the failure of survivors to identify the hidden visual targets (0/28) does not provide evidence for veridical perception under conditions in which normal sensory access would be expected to be severely limited, and is more parsimoniously consistent with internally generated accounts rather than verified external perception.

### 5.3 Dream neuroscience correlations

The phenomenological mapping between REM sleep architecture and canonical NDEs provides crucial converging evidence for the present hypothesis. Neurobiologically, REM sleep is characterized by robust activation of the amygdala and medial temporal lobe memory circuits, alongside a profound downregulation of the dorsolateral prefrontal cortex (dlPFC) (Hobson, 2009; Walker and van der Helm, 2009). This specific network configuration—heightened emotional and associative processing stripped of rational, executive constraint—directly mirrors the hyper-associative nature of NDEs.

Furthermore, research on sleep-dependent memory consolidation demonstrates that the brain does not process daily events indiscriminately. Through the “dream-lag effect,” the brain selectively prioritizes “Personally Significant Events” (PSEs) bound by high emotional intensity, gradually integrating them from hippocampal storage into long-term neocortical networks over a 7-day period (Blagrove et al., 2011). In the context of the dying brain, the rapid failure of prefrontal inhibition unmasks this exact amygdala-driven selection mechanism. Consequently, the panoramic “life review” and the vivid emotionality reported in NDEs are not mystical anomalies, but the accelerated, hyper-associative manifestations of the brain’s established, survival-oriented memory consolidation mechanisms operating under acute metabolic crisis.

### 5.4 Psychedelic-NDE parallels

Beyond sleep architecture, the administration of classic serotonergic psychedelics provides another vital neurobiological analogue. Placebo-controlled studies utilizing intravenous N,N-Dimethyltryptamine (DMT) have demonstrated striking phenomenological overlaps with canonical NDEs. Subjects frequently report ego dissolution, feelings of transcending the physical body, entering alternative realities, and profound alterations in the subjective perception of time (Timmermann et al., 2023). Mechanistically, classical psychedelics act primarily via 5-HT2A receptor agonism, which leads to the disintegration of canonical hierarchical networks—such as the default mode network (DMN)—and a consequent surge in global, cross-network functional connectivity and network entropy.

Within the computational framework of predictive processing, this 5-HT2A-mediated cascade relaxes the precision weighting of high-level priors, permitting bottom-up prediction errors and intrinsically generated affective imagery to overwhelm conscious awareness (Koch et al., 2016). This neurodynamic shift strongly parallels the terminal disinhibition proposed in the Dying-Moment Dream Hypothesis.

However, it is crucial to emphasize that phenomenological similarity does not equate to mechanistic identity. While DMT-induced states frequently feature abstract, kaleidoscopic geometries and encounters with “alien” or “machine-elf” entities, authentic NDEs are overwhelmingly biographical, featuring culturally recognized religious figures, deceased family members, and personal moral evaluations. This divergence clearly indicates that while a severe metabolic crisis may induce an endogenous neuromodulatory shift that mimics psychedelic disinhibition, the specific narrative architecture of the final dream is uniquely scripted by the individual’s own autobiographical memory and cultural conditioning, rather than by neurochemistry alone.

### 5.5 Hospice end-of-life vision research

Research in palliative care settings indicates that End-of-Life Dreams and Visions (ELDVs) are highly prevalent, with conservative estimates suggesting that 50 to 60% of conscious dying patients experience them in the days or weeks preceding death. The content of these visions exhibits remarkable thematic consistency; studies reveal that up to 79% of ELDVs predominantly feature deceased relatives, friends, or spiritually significant figures (Brayne et al., 2006). These figures typically communicate themes of comfort, reassurance, or preparation for a journey.

Unlike the fragmented and distressing hallucinations characteristic of ICU delirium, ELDVs are overwhelmingly perceived as lucid, deeply meaningful, and highly comforting by the patients, often resulting in a profound reduction in the fear of dying. Within the Dying-Moment Dream framework, these hospice visions represent the early, conscious onset of the brain’s emotionally driven generative simulation. Driven by the amygdala’s need to seek secure attachments under conditions of ultimate biological threat, the brain actively constructs these comforting narratives, serving a critical evolutionary and neuroprotective function to mitigate terminal death anxiety.

### 5.6 Cultural phenomenology and ontological collapse

A central prediction of the Dying-Moment Dream Hypothesis is that if afterlife visions are internally generated psychological constructs rather than literal transitions to an objective metaphysical realm, the phenomenological content of these experiences should strictly covary with the experiencer’s culturally acquired semantic memory and theological priors (Klemenc-Ketiš and Kersnik, 2015). Cross-cultural empirical analyses provide substantial support for this prediction, demonstrating structural invariants (reflecting universal neurophysiology) alongside profound, culture-dependent symbolic variability.

In Western NDEs, heavily influenced by Judeo-Christian motifs, experiencers frequently report traversing a dark tunnel toward a personalized “Being of Light” with whom they communicate and feel a profound sense of love. This is often followed by a panoramic life review, reflecting the cultural expectation of a “Last Judgment,” and visions of heaven structured as “cities of light” or pearly gates (Greyson, 2014; Ohkado and Greyson, 2014).

However, these supposedly universal motifs change radically in East Asian contexts. Ohkado and Greyson (2014) demonstrated that in Japanese NDEs, the tunnel motif is virtually absent. While Japanese experiencers perceive an intense light, they do not personify it as a “Being” and do not report communication with it, reflecting a highly secular or Shinto-Buddhist background. Furthermore, the transition border is typically perceived as the “Sanzu River” rather than a tunnel or gate, and heaven is envisioned as soft, infinite flower gardens. Crucially, the life review is missing in contemporary Japanese accounts, correlating perfectly with a societal loss of belief in the Buddhist judge of the dead (Enma-Daio).

Similarly, Theravada Buddhist NDEs in Thailand prominently feature “Yamadutas” (messengers of the death god Yama) rather than loving deceased relatives, and often include terrifying tours of hell and strict karmic judgment (Murphy, 2001). In India, Hindu NDEs are characterized by the “mistaken identity” motif, where the soul is taken by Yamdoots to a cosmic accountant (Chitragupta) who checks a ledger, realizes the wrong person was brought, and sends the individual back to their body (Pasricha, 2008).

These stark iconographic contrasts—summarized in Table 2—provide compelling evidence for psychological constructivism. The dying brain utilizes a universal physiological trigger (hypoxia-induced disinhibition) but filters it through distinct cultural “software,” demonstrating that the final dream is constructed from the individual’s specific semantic and affective archives rather than an objective cosmic geography.

**Table 2:** Cross-cultural iconographic variations in near-death experiences.

Cultural context	Final border/transition	Welcoming figures	Judgment/life review	Image of heaven/peace
Western (Christian/Secular)	Dark tunnel, gate, door	Personalized “being of light,” deceased loved ones	Panoramic life review, self-judgment	Cities of light, glowing clouds
Japanese (Shinto/Buddhist)	Sanzu river	Ancestors (light is seen but not personified)	Generally absent in contemporary accounts	Soft, infinite flower gardens
Thai (Theravada Buddhist)	Road, walking journey (tunnel is rare)	Yamadutas (messengers of the death god Yama)	Strict judgment of karmas (good/bad deeds)	Temples, celestial tiers with jewels
Indian (Hindu)	Riding a cart, walking	Yamdoots, Chitragupta (“man with the book”)	Bureaucratic checking of records; “mistaken identity”	Appearing before religious figures

## 6 Discussion

### 6.1 Competing explanatory models

**Figure 3:**
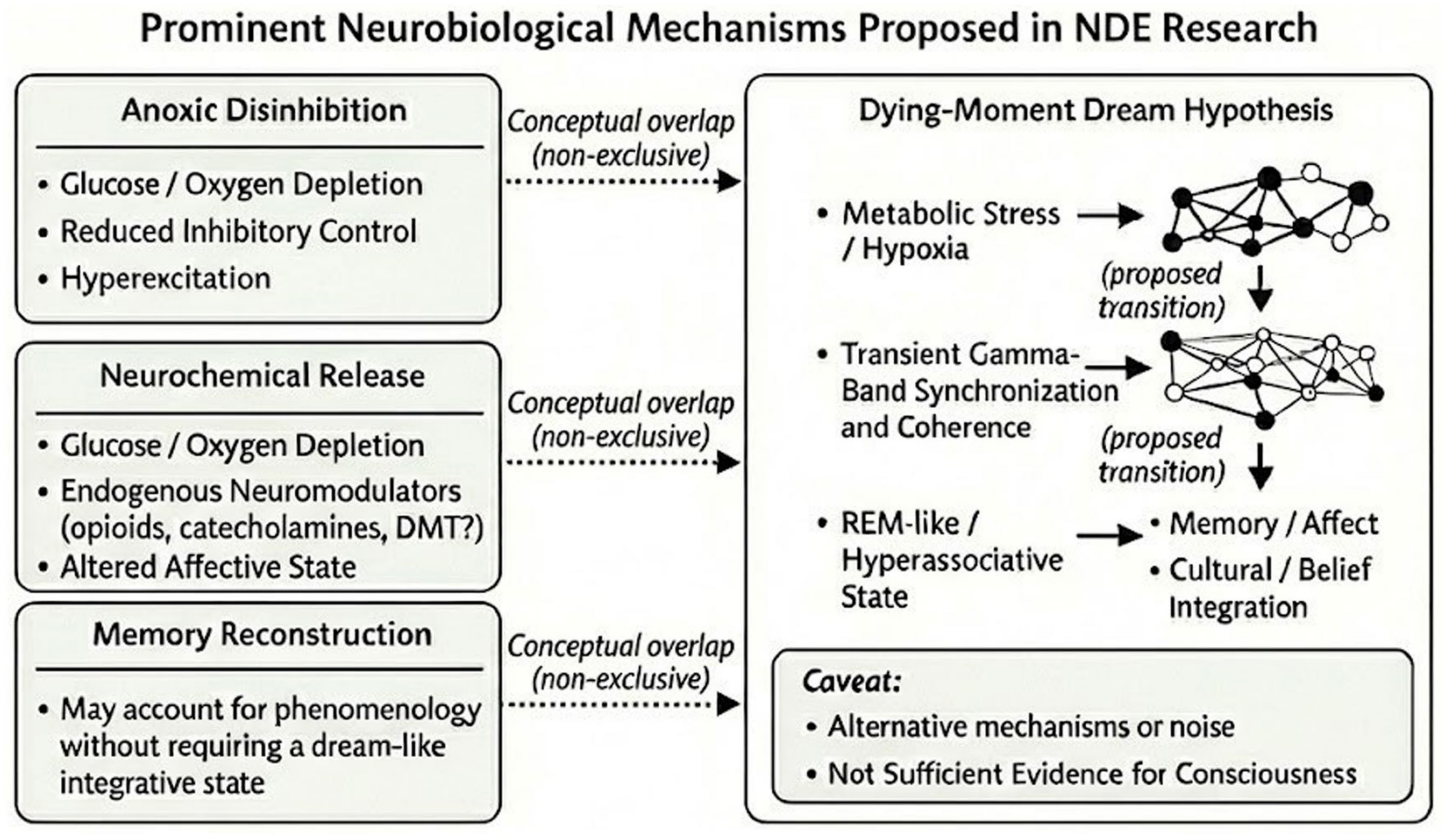
Summary of contrasting neurobiological models of near-death experiences and the dying-moment dream hypothesis. Distinct existing frameworks include metabolic disinhibition (reduced inhibition), neurochemical release (e.g., opioids, catecholamines, debated DMT contribution) resulting in altered affective states, and memory reconstruction. The present hypothesis proposes a transition where transient gamma synchronization under extreme metabolic stress may yield REM-like hyperassociative states shaped by memory, affect, and culturally learned schemas.

The Dying-Moment Dream Hypothesis must be contextualized against existing explanatory models of NDEs. Traditional “hypoxia-only” models argue that NDEs are simply the byproduct of oxygen deprivation and hypercapnia. However, severe hypoxia typically produces fragmented confusion, agitation, and cognitive impairment, which fails to account for the highly structured, lucid, and memorable narratives characteristic of authentic NDEs. Conversely, the “post-resuscitation reconstruction” hypothesis suggests that NDEs are false memories constructed by the brain upon waking as it attempts to make sense of the missing time. This model is increasingly challenged by prospective data (such as the AWARE-II findings) that detect real-time electrocortical biomarkers of cognitive activity occurring simultaneously with the cardiac arrest. The present hypothesis bridges these extremes by positing that hypoxia serves merely as the physiological trigger that initiates cortical disinhibition, while the structured narrative is driven by intact limbic-memory circuits operating in a closed-loop, REM-like state (see Figure 3 for a comparative summary of competing neurobiological models).

### 6.2 Theoretical falsifiability and limitations

A primary limitation of this theory is the inherent impossibility of obtaining real-time subjective phenomenological reports from patients precisely at the point of irreversible death. Furthermore, human evidence for endogenous DMT surges remains indirect, and distinguishing integrated gamma synchrony from purely pathological excitotoxic noise remains a neurophysiological challenge.

To function as a robust scientific theory, this hypothesis must be highly vulnerable to empirical disconfirmation. The model would be significantly weakened or completely falsified if future research demonstrates: (1) an absence of organized peri-arrest network integration (e.g., high-density EEG showing purely stochastic, unstructured electrical noise during the reported experience window); (2) cross-cultural invariance independent of belief exposure (e.g., culturally isolated subjects experiencing highly specific, foreign religious motifs); or (3) no correlation between an individual’s lifelong emotional salience/moral conflict and the affective valence of their subsequent NDE.

## 7 Testable predictions and falsifiability conditions

To further operationalize and test the Dying-Moment Dream Hypothesis, the following measurable predictions and falsifiability criteria are proposed.

### 7.1 Standardized measurement instruments

Future prospective studies must differentiate authentic NDEs from pharmacological hallucinations, ICU delirium, or generalized organic brain syndromes. This requires the mandatory application of standardized diagnostic instruments, primarily the 16-item Greyson NDE Scale (Greyson, 1983)—which serves as the gold standard for reliably differentiating NDEs from simple confusional states due to its high internal consistency and validated threshold scores—and the Life Changes Inventory-Revised (LCI-R) to measure profound, long-term psychological transformations. Crucially, these subjective psychometric evaluations must be rigidly time-locked with high-density quantitative EEG (qEEG) algorithms, specifically monitoring for absolute gamma power surges (>25 Hz) and elevated NSTE directed connectivity thresholds within posterior hot zones.

### 7.2 Operationalized predictions

1. *Neurodynamic signatures*: terminal continuous EEG recordings are predicted to demonstrate quantifiable increases in absolute gamma power (25–55 Hz) within the posterior hot zones, coupled with statistically significant phase-amplitude coupling (crPAC) between gamma and theta/alpha bands, and elevated top-down directed connectivity (NSTE), distinguishing the state from purely stochastic excitotoxic noise.
2. *Affective profiling*: psychometric assessment using the Greyson NDE Scale is hypothesized to reveal that individuals with documented high-intensity emotional trauma, severe anxiety, or unresolved moral conflict exhibit a statistically significant increase in the frequency of distressing or “hellish” NDE profiles compared to baseline populations.
3. Cultural covariance: cross-cultural qualitative coding of NDE narratives is predicted to demonstrate that core structural features (e.g., the nature of transition borders, specific judgment figures) rigidly covary with the experiencer’s culturally acquired semantic memory, precluding the existence of true cross-cultural iconographic invariance.
4. Neuromodulatory overlap: functional neuroimaging of exogenous psychedelic states (e.g., DMT) is expected to show overlapping DMN disintegration with the peri-arrest state, but is predicted to differ significantly in the activation of hippocampal-driven autobiographical memory retrieval networks.
5. Hospice trajectories: longitudinal hospice studies utilizing validated phenomenological mapping are hypothesized to reveal a progressive, measurable increase in the integration of “Personally Significant Events” (PSEs) and deceased attachment figures in end-of-life visions as physiological decline advances.

### 7.3 Conditions for empirical falsification

The hypothesis rests on the premise that NDEs are internally generated, neurobiologically mediated simulations. The framework would be directly falsified under the following empirical conditions:

- *Veridical perception under constrained sensory access*: the hypothesis asserts that the dying brain constructs a closed-loop simulation disconnected from external physical reality. If a patient under continuous clinical observation, particularly during a state of apparent isoelectric scalp EEG (acknowledging scalp EEG detection limits), objectively and accurately reports hidden visual or auditory targets in the resuscitation room (such as the upward-facing visual targets employed in the AWARE-II protocols) that are impossible to perceive via normal sensory pathways or reconstruct via prior knowledge, the “internal simulation” hypothesis would be fundamentally undermined.
- *Cognitive continuity during absolute cortical silence*: the model posits that the final dream is fueled by transient high-frequency activity (e.g., gamma surges, crPAC). If future continuous MEG or deep-brain recordings definitively prove an absolute cessation of all cortical and subcortical oscillations, yet a patient subsequently reports a highly organized, temporally sequential conscious state that can be conclusively time-stamped to the period of absolute neural silence, the neurodynamic foundation of this hypothesis is falsified.

## 8 Conclusion

If the Dying-Moment Dream Hypothesis holds, afterlife-like narratives may not reflect literal transitions to external cosmic destinations, but rather internal psychological landscapes that crystallize into a final conscious simulation. In this view, the afterlife is interpreted not as an objective place, but as a phenomenological process—one constructed from all that a person has felt, believed, feared, and loved. Because there is no awakening to retroactively falsify the simulation, this terminal state is hypothesized to be subjectively experienced as temporally unbounded within the constraints of the final neural event.

## Data Availability

The original contributions presented in the study are included in the article/supplementary material, further inquiries can be directed to the corresponding author.
